# Neoadjuvant therapy for sarcomatoid carcinoma of the pancreas: a case report and review of the literature

**DOI:** 10.1186/s13256-023-04006-9

**Published:** 2023-07-13

**Authors:** Hui Qiu, Chun-Yi Hao

**Affiliations:** Key Laboratory of Carcinogenesis and Translational Research (Ministry of Education, Beijing), Department of Hepato-Pancreato-Biliary Surgery, Peking University Cancer Hospital and Institute, Beijing, People’s Republic of China

**Keywords:** Neoadjuvant therapy, Sarcomatoid carcinoma of the pancreas

## Abstract

**Background:**

Sarcomatoid carcinoma of the pancreas (SCP) is a rare type of malignant pancreatic neoplasm, and its prognosis is even worse than that of conventional pancreatic ductal adenocarcinoma (PDAC). Currently, there is no standard regimen for treating SCP, and the impact of systemic therapy on the survival of patients with SCP has not been well defined.

**Case presentation:**

Herein, we report a 38-year-old Asian man diagnosed of local unresectable SCP with supraclavicular lymph node metastasis, radical excision after camrelizumab and anlotinib therapy, which resulted in a remarkable reduction in the size of primary tumor and complete remission of the metastatic lymph node.

**Conclusions:**

This is the first report of the use of immunotherapy and anti-angiogenesis therapy in a patient with SCP, which provides optimistic data to support the synergistic effect.

## Introduction

Sarcomatoid carcinoma of the pancreas (SCP) is classified as an undifferentiated carcinoma of the pancreas, based on the World Health Organization (WHO) histological classification of exocrine pancreatic tumors. In typical cases, SCPs are composed of cells with spindle cell morphology, with or without a glandular component, and both mesenchymal and epithelial markers are positive in the sarcomatoid component [1]. SCP is a rare variant of pancreatic neoplasms. Blair *et al.* [2] reported a series of 7009 patients, who underwent pancreatic resection over a 25-year period, of which eight patients were diagnosed with SCP on final histopathology with an incidence of 0.11%.

The prognosis of SCPs is worse than standard PDAC, with a median OS of 6 months. Despite this poor result, radical resection still offers chance for cure. The clinicopathological features of the previously reported cases [2–12] and our case are summarized in Table 1. There is no consensus on adjuvant or neoadjuvant therapy for the treatment of SCP. Only one patient received neoadjuvant radiation and gemcitabine plus capecitabine chemotherapy, followed by total pancreatectomy, and was reported to be alive 3 months after the operation. However, the effectiveness of neoadjuvant therapy was not described in detail. Six patients received several different adjuvant treatments (4 gemcitabine-based chemotherapy ± radiotherapy, 1 capecitabine chemotherapy and 1 radiotherapy), including the three patients achieving long-term survival, which suggested that adjuvant therapy may be effective.

**Table 1:** Summary of clinicopathological characteristics of sarcomatoid carcinoma of the pancreas

Author	Year	Age	Gender	Tumor size (cm)	Surgery	Followup (Mo.)	Neo	Adj	Outcome
Uenishi(3)	1999	56	M	11	Y	2	No	No	Die
Michiyo(4)	1999	74	M	4.5	Y	3	No	No	Die
Riva [5]	2006	72	F	NA	No	9	No	No	Die
Ren [6]	2013	48	M	10	Y	28	No	Oxa. Gem. Flu	Alive
Kane [7]	2014	85	M	3.3	Y	26	No	No	Alive
Blair [2]	2017	67	F	4	Y (R2)	2	No	No	Die
		80	F	5	Y	1	No	No	Alive
		63	F	5.7	Y (R1)	1	No	No	Die
		56	F	5	Y	3	Gem. Cap. Radi	Cap	Alive
		79	M	4	Y	3	No	No	Die
		54	M	3	Y	61	No	Gem. Cap. Radi	Alive
		65	M	15	Y	3	No	No	Die
		73	F	9	Y	188	No	Radi	Alive
Abe [8]	2016	74	F	12	Y	8	No	No	Die
Xie [9]	2018	63	M	2.5	Y	18	No	No	Die
Zhou [10]	2019	59	M	2.5	Y	6	No	No	Die
Bukhari [11]	2019	64	M	2.4	Y	19	No	Gem	Alive
Kimura [12]	2020	58	M	5	Y	120	No	Gem	Alive
Present	2022	38	M	7.3	Y	9	Anlo. Camre	Nab. Gem	Alive

*Mo* months, *Neo* neoadjuvant therapy, *Adj* adjuvant therapy, *Gem* gemcitabine, *Oxa* oxaliplatin, *Flu* fluorouracil, *Cap* capecitabine, *Radi* radiation, *Anlo* anlotinib, *Camre* camrelizumab, *Nab *nab-paclitaxel

## Case

**Fig. 1:**
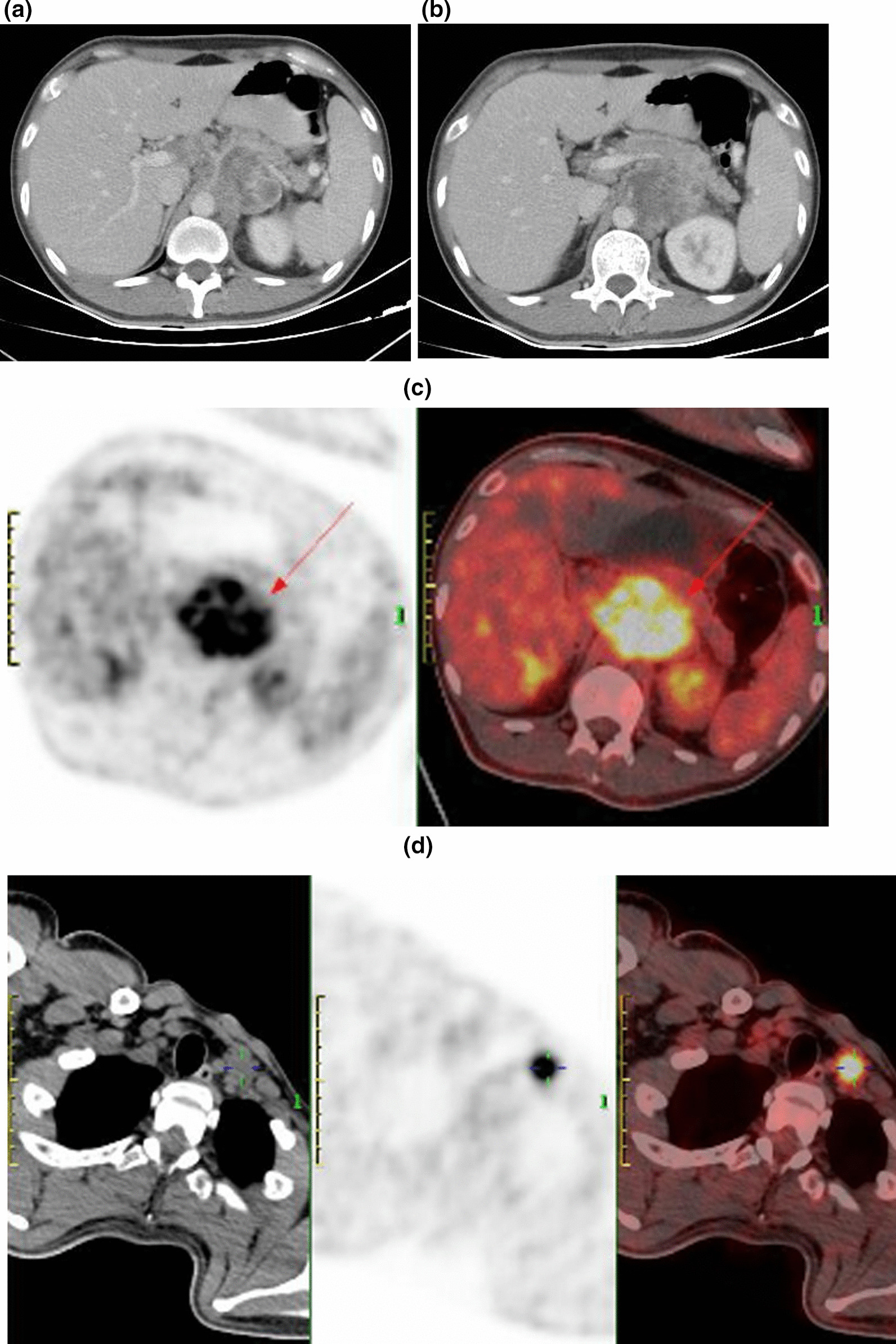
**a**, **b** Initial Computed Tomography scan showing a 73-mm sized mass in the pancreas body and tail with celiac axis (CA) and superior mesenteric artery (SMA) invasion. **c**, **d** Initial 18FDG Positron Emission Tomography/Computed Tomography (PET/CT) scan showing FDG accumulation both in the primary tumor (SUVmax 12.1) and supraclavicular lymph node metastasis (SUVmax 8.8). Arrow indicates the location of the primary tumor

**Fig. 2:**
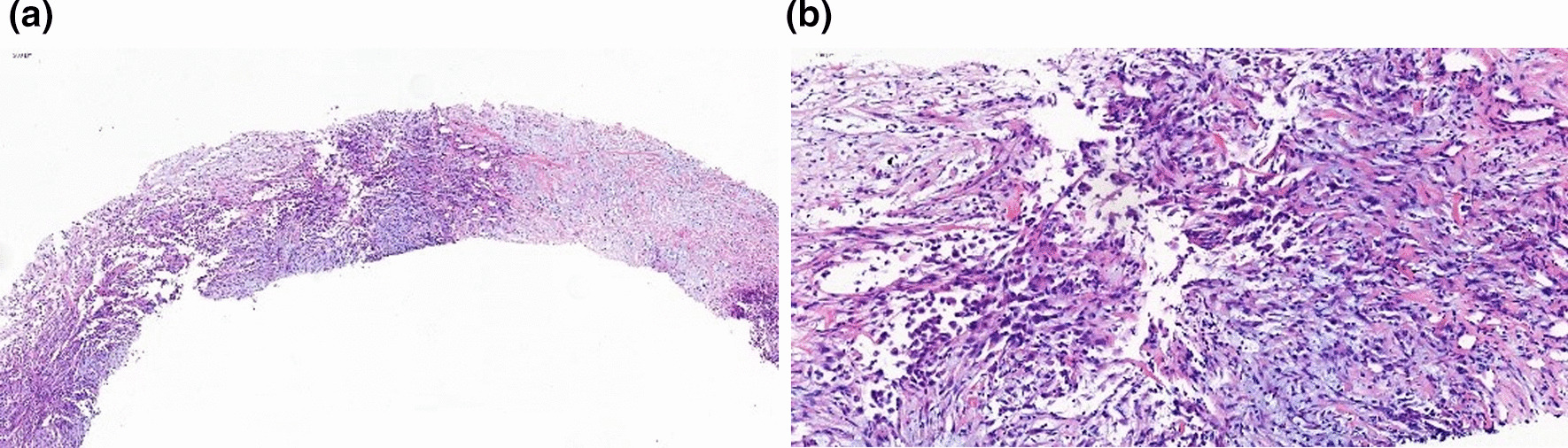
**a**The bioptic specimen showed the tumor was composed of spindle cells. **b** The tumor cells had elongated cytoplasm and hyperchromatic round, ovoid or short spindle-shaped nuclei

**Fig. 3:**
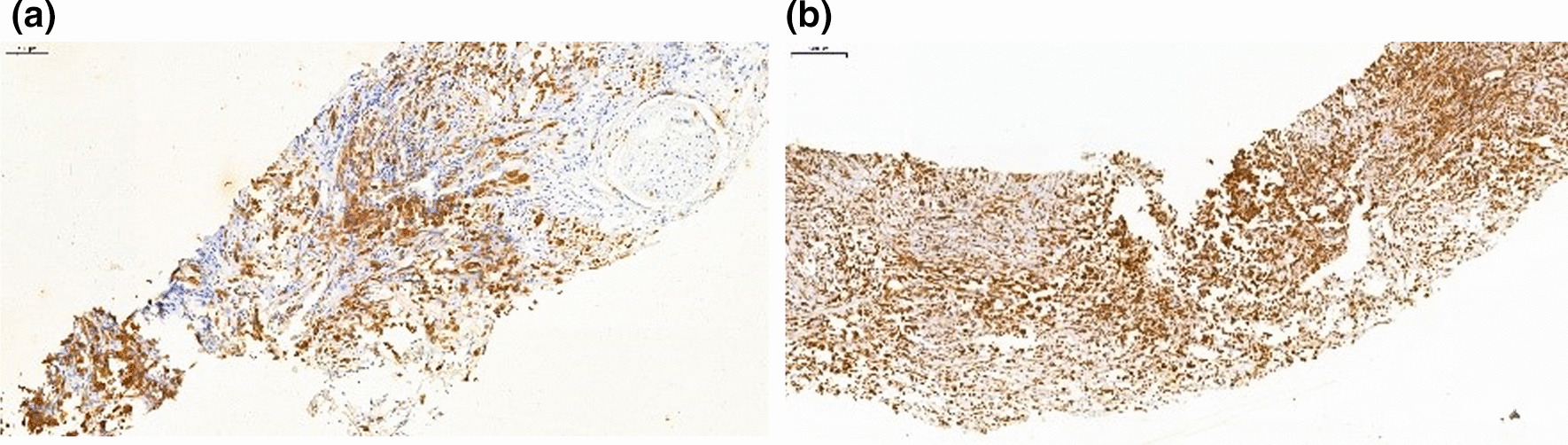
**a** Immunohistochemical examination showed the tumor cells were positive for CK and (**b**)Vimentin

A 38-year-old Asian man was admitted to our hospital for worsening back pain over a course of three months. Physical examination showed tenderness in the epigastrium, and his Eastern Cooperative Oncology Group (ECOG) performance status was 0. Laboratory data revealed that the complete blood count, liver function tests, biochemistry and serum tumor marker levels were within normal ranges. Contrast-enhanced CT identified a solid tumor measuring 73 mm in diameter in the body of the pancreas, with infiltration of the celiac axis and superior mesenteric artery (Fig. 1a–d). 18FDG Positron Emission Tomography/Computed Tomography (PET/CT) scan showed FDG accumulation both in the primary tumor, with a maximum standardized uptake value (SUVmax) of 12.1, and supraclavicular lymph node metastasis with a SUVmax of 8.8. CT-guided fine-needle aspiration revealed high grade malignant neoplasm with sarcomatous spindle-shaped cells (Fig. 2a, b). Based on the immunohistochemical examination which showed that the spindle cells were positively stained for both cytokeratin and vimentin (Fig. 3a, b), thus a pathological diagnosis of SCP was highly suspected. Because there is no standard regimen for treating SCP, modified FOLFIRINOX (5-fluorouracil, irinotecan, and oxaliplatin) was recommended by a medical oncologist. However, the patient was highly concerned about the potential adverse reactions and refused chemotherapy. Therefore, the tumor biopsy specimen were detected by whole-exome sequencing with mean depth of 1523X. Five somatic mutations, IMP4 p.M114I (3.51%), NANOG p.S220T (5.68%), RHPN2 p.H351Q (3.74%), SMARCB1 p.P188Hfs*16 (4.51%) and ZNF91 p.F520C (3.34%) were identified, and we did not detect any known pathogenic or likely pathogenic germline mutation. Moreover, the patient had an expression of PD-L1 (TPS = 5%, CPS = 7) and exhibited a TMB of 0.15 muts/Mb with MSS.

**Fig. 4:**
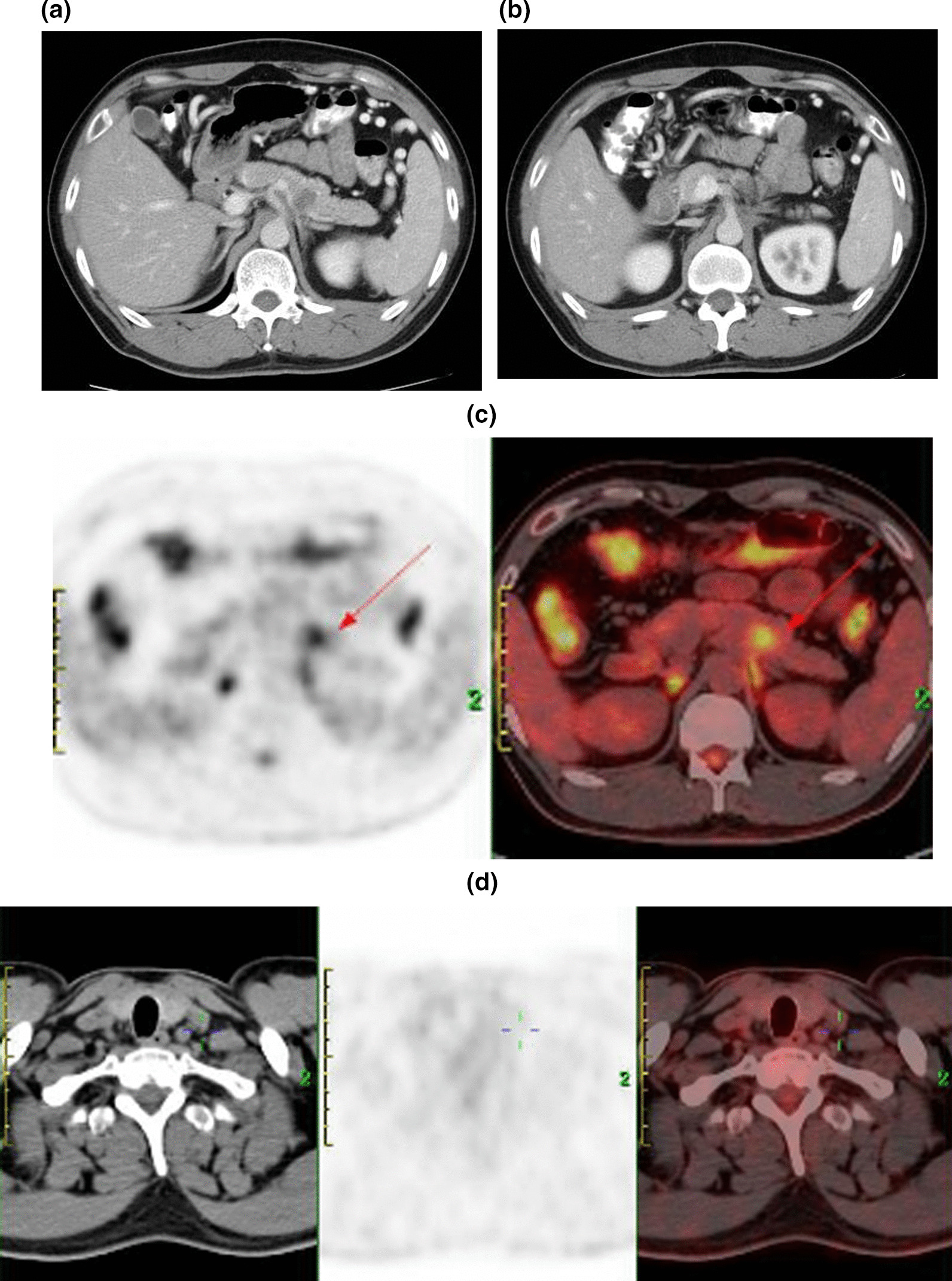
**a**, **b** Computed Tomography scan after administration of camrelizumab and anlotinib, showing decreased size of primary tumor. **c**, **d** Positron Emission Tomography/Computed Tomography (PET/CT) scan showing a significantly decrease of FDG uptake for primary tumor (SUVmax of 4.9) and a complete response in supraclavicular lymph node. Arrow indicates the location of the primary tumor

**Fig. 5:**
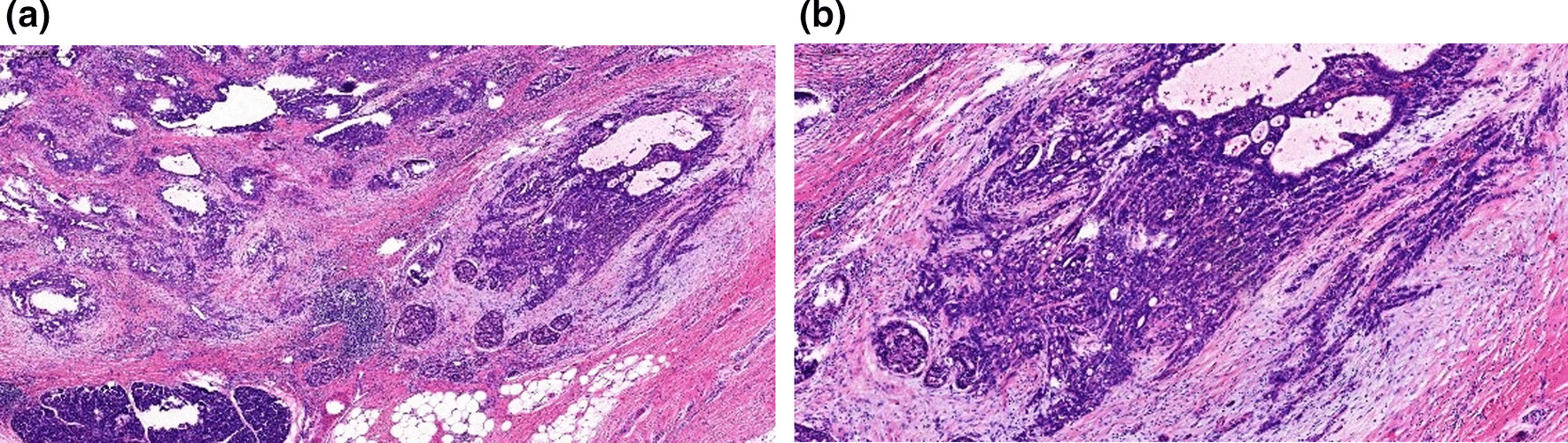
**a**, **b** Microscope analysis showed a poorly differentiated tubular adenocarcinoma without sarcomatoid features (hematoxylin and eosin)

The patient received intravenous camrelizumab 200 mg every 3 weeks plus oral anlotinib 12 mg once daily (2 weeks on/1 week off). After 8 cycles (6-month course) of targeted and immunotherapy, an abdominal CT and 18FDG PET/CT examination showed a significantly decrease for primary tumor both in size and FDG uptake (20 mm in diameter and SUVmax of 4.9), and a complete response in supraclavicular lymph node was observed (Fig. 4a–d). Two weeks after the last dose, the patient underwent a distal pancreatectomy with left adrenalectomy. Microscopically, after the treatment, the tumor was consistent only with poorly differentiated ductal adenocarcinoma, while the sarcomatoid features disappeared (Fig. 5a, b). The tumor was confined to the body of the pancreas without invasion into the adrenal gland. All surgical margins were clear. Lymphovascular invasion and perineural invasion were both identified. Seven lymph nodes were examined and none of them were involved. The tumor was pathologically staged at pT2N0. Immunohistochemical examination showed that the tumor was only positively stained for cytokeratin. The postoperative course was uneventful, and the patient completed a 4-month course of adjuvant treatment of gemcitabine plus nab-paclitaxel (6 cycles). At 9 months post-surgery, he was in good health with no signs of recurrence.

## Discussion

Sarcomatoid carcinoma (SC) can arise in the lung, kidney and gastrointestinal system infrequently. In most cases, the presence of sarcomatoid differentiation indicates highly aggressive behavior and poor prognosis. Seventy percent of small intestine SC patients died of disease within 2 months to 3 years of diagnosis, and 79% had metastatic or recurrent disease at death [13]. Forty percent of SC of the kidney was at advanced stage at operation and died after an average postoperative survival of 8 months [14], leading to a conclusion that operative treatment appears to be ineffective in modifying the behavior of the SC [15]. In this case, we reported a sarcomatoid carcinoma occurring in the pancreas.

The process of epithelial-mesenchymal transformation (EMT) play an important role in the formation of SCP, so both mesenchymal and epithelial markers should be positive in the sarcomatoid component of SCP [1], by which can be distinguished from pancreatic carcinosarcoma (PCS). However, in some previously published reports, the two terms have been used interchangeably, and some reports inappropriately included carcinosarcoma in their reviews of SCP. We re-evaluated previously reported cases and searched English literature on MEDLINE to date. A total of 18 cases of SCP were retrieved from 11 articles. SCP is exceedingly rare with a median OS of 6 months. Most authors thought that the prognosis of SCP is worse than that of standard PDAC. However, Alex and colleagues reported a series of 8 SCP cases in their institution, of which R0 resection was achieved in six patients (75%) and two (25%) experienced long-term survival (> 5 years), similar to the survival rates of standard PDAC. In this case, the patient was presented with locally unresectable disease and distant metastasis, with an expected survival time no more than 3 months. Fortunately, after a successful conversion therapy, he received the surgery, and has been alive for more than 15 months from diagnosis. Therefore, we hold the opinion that radical resection still remains the mainstay of treatment of SCP, and effective systematic therapy can bring benefits to patients. The diagnosis of SCP should not portend to a necessarily dismal prognosis and still offers a chance for cure.

Tumor grade, lymph node status, MIB-1 labeling index and adjuvant systemic therapy were considered the prognostic factors of SCP in some reports [2, 6, 11, 12]. Gemcitabine-based chemo or chemoradiotherapy regimen was most commonly used. Neoadjuvant therapy in the setting of SCP remains uncertain, because the objective response rate (ORR) of systemic therapy was unclear. Only one patient received neoadjuvant chemoradiotherapy in the literature, but the effects of treatment had not been described in detail. In present case, after anlotinib and camrelizumab being used, the primary tumor was significantly reduced and the metastatic lymphnode disappeared, which, to our knowledge, was the first report of the use of targeted and immunotherapy in a patient with SCP successfully.

Mutations of *SMARCB1* gene, which considered as a core subunit, was detected in this case, and the mutation site is located in the 2X tandem repeat region, HIV-1 integrase-binding region, MYC binding region, 1 repeat region. This gene variant was not included in the OncoKB, ClinVar, COSMIC database. *SMARCB1* mutation may result in incomplete expression and loss of function of the SMARCB1 protein, which may be regarded as an oncogenic mutation. *SMARCB1* mutations have been detected in rhabdomyoma, familial schwannomatosis, small cell hepatic fibroblastoma, skeletal extracellular myxoid chondrosarcoma, undifferentiated sarcoma, epithelial sarcoma, meningiomas and poorly differentiated chordomas. Although the mechanism of *SMARCB1* deletion leading to malignant transformation remains unclear, expression analysis suggests that deletion of SMARCB1 activates gene expression programs associated with proliferation and dedifferentiation. *SMARCB1* mutated cells were found to be dependent on EZH2, a component of PRC2, and EZH2 inhibitors were tested with SMARCB1 mutated cancer patients in clinical trials. There are no targeted drugs targeting the *SMARCB1* approved by FDA/NMPA or recommended by NCCN guidelines in SCP currently [16–19].

Due to personal refusal to chemotherapy, we tried administering camrelizumab in combination with anlotinib to the patient. Anlotinib is a small molecule multi-targeted tyrosine kinase inhibitor, which strongly inhibits many targets such as vascular endothelial growth factor receptor, platelet-derived growth factor receptor, fibroblast growth factor receptor, and c-Kit. Preclinical studies have demonstrated that anlotinib has potent antiangiogenic activity through inhibition of both the VEGF and FGF signaling pathways. Chi proved that anlotinib had broad-spectrum antitumor activity in patients with several metastatic soft tissue sarcoma entities [20]. In another case report of gallbladder SC, obvious tumor necrosis was also observed after treatment with anlotinib [21]. Recently, agents targeting immune checkpoint signaling have shown promising results in patients with several malignancies. Camrelizumab is an anti-programmed death (PD-1) mAb which was also given to the patient at the same time, because the patient had an expression of PD-L1 (TPS = 5%, CPS = 7), which has been observed commonly in SC patients. Nicola [22] reported that PD-L1 CPS ≥ 1 was common in SCPs (5/6) with half samples expressing PD-L1 CPS ≥ 50, and Vieira [23] proved that PD-L1 overexpression has been observed in over half of pulmonary sarcomatoid carcinomas. Although the response to immune checkpoints inhibitor have not been evaluated specifically in SCP, Charlotte [24]and colleagues found that in patients with lung SC who underwent nivolumab treatment, the objective response rate was 58.8% with PD-L1 + and 0% with PD-L1-(*p* = 0.44), indicating a trend toward higher PD-L1 expression in responsive diseases. Ultimately, this patient achieved a favorable outcome after targeted therapy plus immunotherapy, followed by a radical surgery.

## Conclusion

Sarcomatoid carcinoma of the pancreas is a rare histologic subtype of undifferentiated pancreatic carcinoma. The clinical course of our patient confirms that anlotinib and camrelizumab play an important role for patients with SCP. However, no more data are present in literature, our successful treatment of this rare SCP patient provides optimistic data to support the synergistic effect of immunotherapy and anti-angiogenesis therapy. This combination therapy shows efficacy in terms of resectability and overall survival. More studies are needed on larger population regarding this proposed treatment.

## Data Availability

Some or all data, models, or code generated or used during the study are available.
